# Severe Legionnaires’ disease

**DOI:** 10.1186/s13613-024-01252-y

**Published:** 2024-04-02

**Authors:** Jordi Rello, Camille Allam, Alfonsina Ruiz-Spinelli, Sophie Jarraud

**Affiliations:** 1 Global Health ECore, Vall d’Hebron Institut of Research (VHIR), Barcelona, Spain; 2 Formation Recherche Evaluation (FOREVA) Research Group, CHU Nîmes, Nîmes, France; 3 Institut des Agents Infectieux, Centre National de Référence des Légionelles, Hospices Civils de Lyon, Lyon, France; 4 Centre International de Recherche en Infectiologie (CIRI), Équipe Pathogenèse des Légionelles, Université Lyon, Inserm, U1111,Université Claude Bernard Lyon 1, CNRS, UMR5308,École Normale Supérieure de Lyon, Lyon, France; 5 Medicine Department, Universitat Internacional de Catalunya, Barcelona, Spain; 6 Centre National de Reference des Légionelles, Institut des Agents Infectieux, Hospices Civils de Lyon, 103 Grande rue de la Croix Rousse, 69317 Lyon Cedex 04, France

**Keywords:** Legionellosis, Legionnaires’ disease, Severe community-acquired pneumonia, Biomarkers, Levofloxacin, Macrolides, Acute respiratory distress syndrome, Immunocompromised patients

## Abstract

**Background:**

Legionnaires’ disease (LD) is a common but under-diagnosed cause of community-acquired pneumonia (CAP), although rapid detection of urine antigen testing (UAT) and advances in molecular testing have improved the diagnosis. LD entails intensive care unit (ICU) admission in almost one-third of cases, and the mortality rate ranges from 4% to 40%. This review aims to discuss recent advances in the study of this condition and to provide an update on the diagnosis, pathogenesis and management of severe LD.

**Results:**

The overall incidence of LD has increased worldwide in recent years due to the higher number of patients with risk factors, especially immunosuppression, and to improvements in diagnostic methods. Although LD is responsible for only around 5% of all-cause CAP, it is one of the three most common causes of CAP requiring ICU admission. Mortality in ICU patients, immunocompromised patients or patients with a nosocomial source of LD can reach 40% despite appropriate antimicrobial therapy. Regarding pathogenesis, no *Legionella*-specific virulence factors have been associated with severity; however, recent reports have found high pulmonary *Legionella* DNA loads, and impairments in immune response and lung microbiome in the most severe cases. The clinical picture includes severe lung injury requiring respiratory and/or hemodynamic support, extrapulmonary symptoms and non-specific laboratory findings. LD diagnostic methods have improved due to the broad use of UAT and the development of molecular methods allowing the detection of all Lp serogroups. Therapy is currently based on macrolides, quinolones, or a combination of the two, with prolonged treatment in severe cases.

**Conclusions:**

Numerous factors influence the mortality rate of LD, such as ICU admission, the underlying immune status, and the nosocomial source of the infection. The host immune response (hyperinflammation and/or immunoparalysis) may also be associated with increased severity. Given that the incidence of LD is rising, studies on specific biomarkers of severity may be of great interest. Further assessments comparing different regimens and/or evaluating host-directed therapies are nowadays needed.

**Graphical Abstract:**

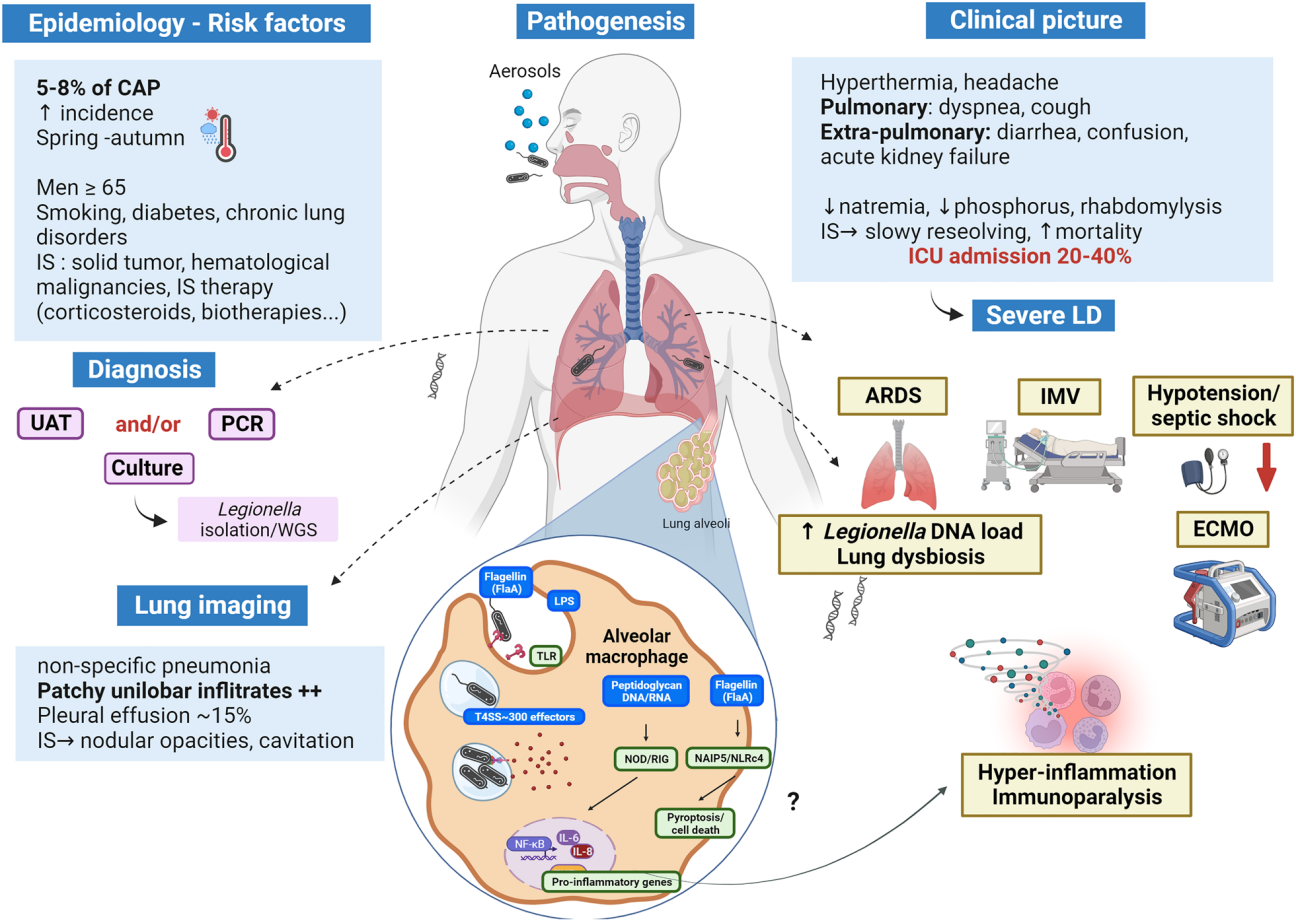

## Introduction

Legionellosis, a waterborne disease, is becoming a major public health problem throughout the world. Although it is considered to be underdiagnosed and underreported, its incidence is rising. Morbidity and mortality remain high, as well as the associated health costs; therefore, early recognition of the disease and appropriate management are mandatory [1, 2]. *Legionella* pneumonia, also known as Legionnaires’ disease (LD), is an important cause of community-acquired and nosocomial pneumonia.

A high proportion of patients with LD requires intensive care unit (ICU) admission for artificial organ support therapy, with a rate that ranges from 20% to 40% according to the study [1, 3–5]. Patients with a history of smoking or chronic lung disease, those over 50 years of age and those with immunocompromising conditions (chronic steroid use, solid organ transplant, solid tumour or haematological disease) have an increased risk of developing LD. Common causes of ICU admission for LD are acute respiratory distress syndrome, septic shock and acute renal failure. The aim of this review is to outline the latest advances in the diagnosis and therapy of severe LD. Considerations regarding infection prevention and control are beyond the scope of this article.

## Epidemiology

*Legionella species* (spp) are Gram-negative bacteria with strict growth requirements, given that between three and 5 days of incubation are required for its colonies to be detectable. Historically, this pathogen has presented a major diagnostic challenge. In humans it has the ability to cause a severe pneumonia, a non-pneumonic disease (generally benign) called Pontiac fever, and extrapulmonary legionellosis [1, 2]. The clinical and radiological presentation of LD is non-specific and can mimic other types of pneumonia. This review focuses on severe LD, although severe extrapulmonary forms of *Legionella* infection may also occur, particularly in immunocompromised patients.

*Legionella* species are located in aquatic habitats, soil, and water distribution systems. They have the ability to survive intracellularly in various protozoans and to proliferate within biofilms which provide additional protection from the environment [3]. More than 65 species of *Legionella* have been recorded, but not all of them are equally responsible for infectious disease. *Legionella pneumophila* (Lp) is the most frequent cause of disease (mostly the serogroup (sg) 1 (Lp1), followed by sg3 and sg6), which account for 80–90% of cases in Europe and in the US [4]. In Australia and New Zealand the predominant species is *L. longbeachae* [5, 6] which accounts for up to 51% of causative agents for LD [6], while Lp represents 31% [5, 6]. This heterogeneous distribution may be attributed to several factors: for example, in New Zealand there is a more systematic use of *Legionella* screening by PCR, and clusters of *L. longbeachae* infections are seen in early spring‒summer and might be linked to increased outdoor activity, such as gardening in the warmer months [6]. However, the exact reason for its predominance remains unknown as *L. longbeachae* strains display a high genetic diversity, probably indicating the presence of multiple sources of infection [7].

Human infection occurs due to the inhalation or aspiration of aerosols containing the pathogen. After inhalation, *Legionella* invades lung alveolar macrophages, inhibiting their bactericidal activity and turning them into a niche for its replication, in a similar way to the mechanisms that it uses to survive within its protozoan hosts [3, 5, 8]. With the exception of a single documented case, human-to-human transmission does not occur [9].

The main sources of contamination are water network systems, spas and cooling towers [10]. Interestingly, *L. longbeachae* disease has been associated with gardening and the use of potting soil, commercial bagged soil, and compost materials containing this bacterium [6, 11], and *L. anisa* with dental practices [12]. Risk factor analysis reveals that temperatures > 20 ℃ are a significant risk factor for *Legionella* colonisation in dental chair equipment. Water-bearing instruments and therapy in dental chair units requiring water-lines generate aerosols which may lead to an infection risk among dental staff and patients. Biofilms in contaminated pipes may generate infection in dialysis units or in hospitalised patients. Regular monitoring of the water quality to identify the presence of significant *Legionella* loads and the application of routine disinfection procedures are recommended to minimise the risk of infection. Furthermore, the maintenance of a minimum temperature for hot water (storage water > 60 ℃, distribution water > 50 ℃) and a maximum temperature for cold water < 20 ℃ [13] is necessary to prevent the multiplication of *Legionella* inside a water network. Episodes of LD must be notified to the public health authorities to identify the source of contamination.

The proportion of Lp among the causative agents of community-acquired pneumonia (CAP) was estimated to be 4.6% in a recent meta-analysis [14], and nearly twice this figure in patients admitted to the ICU. Historically, it has been reported to be one of the three most common causes of severe CAP requiring ICU admission [15–18], although advances in prevention, early detection and early appropriate therapy have reduced its incidence as a cause of ICU admission over the last decade [19, 20]. LD may be a cause of severe hospital-acquired pneumonia [21] but there is no evidence to suggest a role in aspiration pneumonia [22]. In temperate climates, most LD cases occur from late spring to early autumn [23, 24], but in tropical regions they may be recorded throughout the year [25]. A general increase in incidence has been observed in Europe, the US, Canada and Australia in recent years [24, 26, 27]. The reasons for this worldwide increase are not totally elucidated, but it is most pronounced in older age groups (> 60 years) and with a general accentuation of seasonal trends, without any systematic changes in the methods used for its diagnosis in Europe [24]. Many studies have found that increased precipitation, temperature, and relative humidity are positively associated with the occurrence of LD, particularly a sequence of elevated temperatures followed by a period of increased precipitation, high relative humidity, and low wind [28, 29]. The intensification of extreme meteorological events due to climate change (for instance, high temperature variations and heavy rainfall), may create conditions for the development of *Legionella* and increase the incidence of LD [30, 31].

## Pathogenesis of Legionnaires’ disease

Lung histopathological studies of LD in deceased patients have reported that terminal bronchioles are predominantly affected by an extensive intra-alveolar exudation of macrophages, neutrophils, erythrocytes, and fibrin [32, 33]. They also describe an oedema-induced widening of the alveolar septa as a characteristic feature of LD caused by *Legionella* proliferation, mainly in macrophages but also in lung epithelial cells, which can lead to necrotic damage to the lung tissue. The tropism of *Legionella* for alveolar macrophages explains the need for deep pulmonary sampling to optimise bacteriological diagnosis.

The lung cells, in particular macrophages, infected by Lp not only provide intracellular niches that facilitate its pathogenesis, but also contribute to the immune response against it. To establish an infection, Lp uses its type IV secretion system (T4SS), one of the key virulence factors, which translocates approximately 300 effector proteins into the host cell cytosol [34]. These effectors modulate host cell vesicle trafficking and endosomal maturation pathways, thereby inducing bacterial replication. The immune system, in turn, has developed many strategies to recognise intracellular bacteria, leading to a strong inflammatory response to clear the Lp infection. Toll-like receptors (TLRs), transmembrane receptors on the surface of monocytes, macrophages and dendritic cells (DCs) that recognise bacterial ligands such as lipopolysaccharide (LPS) and flagellin take part in the anti-*Legionella* response by activating the transcription factor Nuclear Factor kappa-B (NF-κB), which triggers the production of pro-inflammatory cytokines, such as tumor necrosis factor-alpha (TNF-α), interleukin-8 (IL-8) and IL-6, as well as other chemokines. Other intracellular effectors such as Nucleotide Oligomerization Domain (NOD)-like receptors and Retinoic Acid Inducible Gene I (RIG-I)-like receptors recognise bacterial compounds (double-stranded DNA, peptidoglycan, flagellin) in the cytosol of infected cells. DCs, neutrophils (PNNs) and Natural Killer (NK) cells also play a role in the anti-*Legionella* defense. The multiplicity of these receptors ensures activation of innate immunity even if one of them is dysfunctional [35].

Most of the published data on the anti-*Legionella* immune response are derived from cellular (primary lung cells or immortalised lines) or animal models. Murine models are the most commonly used. However, wild-type mouse strains are naturally non-permissive to *Legionella* infection, due to the impossibility of intracellular replication [36]; only macrophages from A/J mice deficient in the NAIP5 (or Birc1e) pathway of the inflammasome allow an intracellular infection. The NAIP5 pathway is involved in the recognition of flagellin. Therefore, another way to study the infection in the mouse model is to use Lp with non-functional flagellin [37]. Thus, studies in animal models are an imperfect but nonetheless useful reflection of the pathophysiology and the immune response in human infection.

The pathogenesis of severe LD remains poorly understood. Several virulence factors involved at different stages of pathogenesis have been described in the literature [34]. In a recent review focusing on surface-associated and secreted virulence factors, the authors highlighted the role of zinc metalloprotease (ProA), macrophage infectivity potentiator (Mip) and flagellin (FlaA) in virulence regulation, host tissue degradation and immune evasion [38]. In particular, ProA may contribute to bacterial proliferation and dissemination in the human lung, as well as to the formation and progression of lung damage, through a protease extracellular activity against the host lung tissue. Altogether, this may increase pneumonia severity. In clinical practice, no *Legionella* virulence factor is known to be associated with the severity of LD. Studies have shown an association between initial *Legionella* DNA load in respiratory samples and initial high pneumonia severity score, ICU admission or prolonged hospitalisation [39, 40]. The respiratory tract microbiome (RTM) balance is impaired in the case of severe LD. There is a low microbial diversity in patients with invasive mechanical ventilation (IMV) and the presence of opportunistic pathogens (fungi, archaea, and protozoa) seem to contribute to the progress of pneumonia [41]. As the *Legionella* biomass has been found to correlate with disease severity and has been linked to co-morbidities, the quantification of the pathogen should be included among the procedures in patient monitoring. Furthermore, IMV, long hospitalisation periods, and combined antimicrobial therapies alter the lung microbiome [39]. The interaction between the balance of the respiratory microbiome, the dynamics of the pathogen load and the interventions associated with hospitalisation (mechanical ventilation, antibiotics, etc.) plays an essential role in the recovery of patients with pneumonia. Regarding the immune response, the degree of activation of the NF-κB pathway in vitro depends on the Lp isolate [42, 43]; Lp strains that induce higher NF-κB activation in vitro induce greater weight loss, higher mortality, more severe lung inflammation and higher levels of serum cytokine production in mice. In humans, the data are sparse; one study in a small number of patients showed that the intensity of the inflammatory cytokine response was greater in the most severe patients [44]. A recent study of a prospective cohort found that severe LD patients under mechanical ventilation presented an initial increase in the systemic secretion of seven pro-inflammatory mediators and a leukocyte hypo-responsiveness with a lower secretion capacity for 16 cytokines, suggesting immunoparalysis [45].

All *Legionella species* show evidence of long-lasting coevolution with their protozoan hosts [46]. *Legionella* is an opportunistic pathogen that incidentally infects humans. LD is an evolutionary dead-end for *Legionella*; it is either cleared by the immune system or results in the death of the patient. No or very rare commensal status has been reported for *Legionella* and there is no human-to-human transmission, suggesting that any human-specific adaptations that may occur during infection are unlikely to be fixed in the population [47]; this may partly explain the low level of antibiotic resistance.

## Clinical picture

Patients with LD are more likely to develop severe CAP than those with other atypical respiratory pathogens [48]. Risk factors for acquiring *Legionella* pneumonia include smoking (regardless of age) [49], previous chronic lung disorders, diabetes, hematological malignancies or solid tumors under cytotoxic chemotherapy, organ transplant, and immunosuppressive treatment, such as glucocorticoids or TNF-α blockers. Higher rates have been reported in men and in the elderly [1, 50, 51]. There are non-specific clinical symptoms that distinguish LD from other types of pneumonia, but the specificity of clinical and laboratory findings is increased when these parameters are combined. As this infection may present without a clear epidemiological source, it is important to be alert to distinctive features that would suggest the diagnosis. Extrapulmonary clinical manifestations such as gastrointestinal and neurological disorders added to respiratory symptoms, are suggestive of this infection. Specifically, diarrhoea, acute confusion, acute kidney failure and high fever with relative bradycardia are commonly identified; although it can be difficult to distinguish these symptoms from those of septic complications [1, 2].

Though non-specific, some common laboratory findings such as hyponatremia, decreased levels of serum phosphorus, rhabdomyolysis with elevated creatine kinase levels, impaired renal function, microscopic hematuria, hyperleukocytosis with lymphopenia, elevation of serum ferritin and C-reactive (CRP) protein may suggest LD diagnosis [1]. In terms of lung imaging, there are no pathognomonic imaging features for LD; nevertheless, some radiographic and tomographic patterns in combination with the clinical presentation may be suggestive of its presence.

Chest radiographs show pulmonary infiltrates with the most common pattern being a patchy, unilobar infiltrate. Patchy unilobar infiltrates are seen early, with rapid generalisation to bilateral interstitial pneumonia as hypoxemia progresses. Bilateral opacities are present in 50% of adults in intensive care ([52]). Pleural effusion can be found in 15–50% of cases [1, 50]. As in most bacterial pneumonia, it is a parapneumonic effusion. The bacterium is rarely isolated from pleural fluid. The pleural effusion does not usually modify management, because empyema is uncommon, although it is associated with more severe cases in immunocompromised patients [53]. Radiographic findings tend to worsen, particularly during the 1st week, in the setting of clinical improvement. This feature should be borne in mind so as to avoid changing the antibiotic therapy initiated or starting further invasive diagnostic investigations [50, 54]. The consolidations on tomography scans are larger than would be expected based on the radiographs, and are surrounded by ground-glass opacities [50].

In addition, several extra thoracic involvements due to *Legionella* species infection have been described and well-documented, known as extrapulmonary legionellosis, particularly among immunocompromised patients. Within the cardiovascular system this can cause myopericarditis and endocarditis [55–57]; encephalitis, brain abscess or cerebellar ataxia as neurological complications [55, 58, 59]; a gastrointestinal compromise with pancreatitis, colitis, liver and spleen involvement [60]; joint [61] and skin damage (cellulitis, necrotising fasciitis) [62–64]; and disseminated intravascular coagulation, among others. Extrapulmonary forms mainly occur secondary to a pulmonary localization due to the spread of *Legionella* through the blood, even though the primary focus may be outside the lung: for example, single skin infection developing after direct inoculation from the environment [61, 64].

As mentioned above, immunocompromise is an important risk factor for LD, especially impaired cell-mediated immunity. Within this population, the incidence of LD continues to rise and mortality remains high [53, 65, 66]. Unlike the general population, in immunocompromised patients *Legionella* infection may have an unusual clinical presentation; both *L. pneumophila* and *non-pneumophila* species may be responsible for the infection, and present similar outcomes [65, 66]. In a 15-year retrospective study conducted in a large transplant referral center in Seattle, *Legionella spp* were found to be opportunistic pathogens which notably increased patient morbidity and mortality. That study also found LD to be fatal in one-third of transplant recipients despite appropriate treatment [65]. A recent study in a retrospective cohort showed that LD in solid transplant recipients was more severe: 56% of patients were admitted to an ICU, and these more severe patients presented more often negative UAT, lymphopenia and respiratory symptoms [53]. Immunocompromised patients may have different radiological manifestations; nodular opacities may be found [53], and despite appropriate antibiotic therapy, around 10% of cases may cavitate [1, 50].

Among ICU patients, the majority require mechanical ventilation (MV) [67, 68], present multi-organ failure or septic shock [68, 69]. Prognostic factors related to mortality or poor outcome are: age, female sex, kidney failure, prolonged corticosteroid therapy, CRP levels exceeding 500 mg/L[70], high severity-of-illness (APACHE II score > 15 at admission or SAPS II above 46), severe hypoxemia requiring ventilatory support or high-flow nasal oxygen, renal disease, rhabdomyolysis, presence of malignancy, immunosuppression, nosocomial source of infection, and a delay > 24 h in administration of appropriate treatment [58, 71, 72].

LD has an overall mortality of 4–18% but higher rates (close to 40%) are reached in immunocompromised individuals and in those requiring ICU admission [67, 69, 70]. Co-infections have rarely been described for *Legionella*. During the first wave of the COVID-19 pandemic, a co-occurrence of *Legionella* and SARS-CoV-2 was described in 7/49 patients for a period of 1 month [73]. These co-infected patients presented higher severity than patients with LD only. Since then, other severe SARS-CoV-2 and *Legionella* co-infections have been described [74]. Furthermore, Influenza viruses may overlap with LD [75]. The impact of dual infection usually worsens the patients’ prognosis. Some opportunistic bacterial and fungal pathogens such as *Pseudomonas*, *Stenotrophomonas*, *Candida* or *Aspergillus* may also be found as co-infection or superinfection microorganisms [41, 76]. In cases of LD, physicians should consider multiple pathogen infections.

Mortality rates are lower than in the case of pneumococcal pneumonia. This feature is of great significance, as LD has always been associated with higher mortality in patients with comorbidities, but comorbidities actually occur less frequently in patients with *Legionella* pneumonia than in patients with pneumococcal pneumonia [77]. Mortality can be above 30% in patients admitted to ICU with severe hypoxemia or acute respiratory distress syndrome, and prompt, effective therapy has a fundamental role in outcome. Age above 50 years, SOFA scores above 6 and delay in antibiotic therapy active against *Legionella* are significant predictors of 30-day mortality. Multiple reports have documented the association of LD, rhabdomyolysis, and acute kidney injury (AKI). Andrea et al. [67] reported that nearly 80% of adults with LP developed AKI in a 10-year cohort of ICU patients, half of whom required renal replacement therapy. The median SOFA score was 6 and two-thirds required vasoactive agents for septic shock. Delayed initiation of therapy for LD results in worse outcomes, and in some series it has more than doubled ICU mortality [23]. A strategy of test and treat, with early diagnosis and right-first-time therapy, is the cornerstone for optimal management.

## Diagnosis of Legionnaires’ disease

Two methods are of major interest for the early diagnosis of LD: urine antigen detection and molecular study (polymerase chain reaction: PCR) in respiratory specimens. Urine antigen tests (UATs) account for 70–80% of cases diagnosed in Europe and the US, making them the first-line diagnostic test for LD [78]. The detected antigen is a component of the structure of bacterial LPS, and antigenic diversity is the basis for the identification of Lp serogroups. UATs have been developed to detect Lp1 LPS, which is identifiable very early in the course of the disease (from 1 to 3 days after symptom onset) and can persist for several weeks or months [79]. The UAT result is not generally influenced by the prior use of antibiotics. Although limited to Lp1, it is the fastest diagnostic technique available (15–30 min) and thus allows early adaptation of the antibiotic therapy [1, 23]. According to two meta-analyses, UATs have a moderate sensitivity of 70–90% and a specificity of almost 100% for the diagnosis of LD caused by Lp1 [80, 81]. Furthermore, UATs appear to have a higher diagnostic yield in patients with more severe pneumonia; in two large Lp1 outbreaks in Spain and the Netherlands, the sensitivity of UAT was below 50% in patients with mild LD, but above 85% in patients with severe LD [82, 83]. Sensitivity is lower in patients with hospital-acquired LD or in immunocompromised patients, as infections in these populations are more likely to be caused by non- Lp1 or Lp1 strains with a LPS less well-recognised by commercially available tests [84, 85]. These data suggest that LD may be under-diagnosed when only UATs are used.

The Infectious Disease Society of America (IDSA) and the American Thoracic Society (ATS) recommend performing UAT for *Legionella* antigen only in adults with severe CAP (i.e., patients with septic shock, respiratory failure requiring mechanical ventilation, or with three minor severity criteria) or when CAP is associated with a *Legionella* outbreak or recent travel [86]. This policy probably reduces the diagnosis of mild Legionnaires’ Disease. Furthermore, the fact that non-serogroup 1 Lp are not detected by UAT may lead to an underestimation of the total LD incidence. Two large studies conducted in the US before and after the publication of the IDSA/ATS guidelines [86] showed a similar UAT positivity rate of 1.5%, and demonstrated the benefit of positive antigenuria in the management of patients who are not admitted to the ICU and do not always receive probabilistic treatment targeting *Legionella* [23, 87]. According to the IDSA/ATS guidelines, UAT should be used to assess LD and in the presence of non-specific criteria commonly associated with LD, such as hyponatremia and diarrhea. This policy may improve UAT cost-effectiveness.

The detection of *Legionella* DNA by PCR from respiratory tract samples has been increasingly used over the last 10 years [88, 89]. This method has the potential to detect all known *Legionella* species and all sgs (1–16) of Lp. It has good specificity and sensitivity for *Legionella spp* detection, especially when performed on respiratory tract samples [2, 23]. A 2016 meta-analysis showed overall sensitivities and specificities of 97.7% and 98.6% for bronchoalveolar fluids and of 96.8% and 99.4% for sputa [90]. In contrast, urine and serum PCRs have low sensitivities, ranging from 41% to 50% for serum and 14–70% for urine depending on the study [91, 92]**,** which are insufficient for diagnosis. In extrapulmonary forms, PCR can be performed on localised samples (pleural fluid, biopsies, joint fluid, etc.). *Legionella* DNA load in the lung has been shown to be associated with a higher Fine score, more frequent ICU admission and longer hospital stay [39], and was recently shown to be higher in patients under mechanical ventilation [45]. In addition, the maintenance of a positive PCR over time may be associated with the development of a pulmonary abscess or cases of persistent or slowly resolving LD [93, 94]. This suggests that monitoring DNA levels over time may be a good tool for tracking the course of infection.

All test methods have inherent weaknesses for the detection of *Legionella*. Several studies have compared the results of PCR in respiratory specimens with those of UAT [90, 95]. PCR results led to reclassification of 18–30% of LD symptomatic cases with previously negative UAT; conversely, 11–22% of patients confirmed with Lp1 by UAT were negative by PCR [39, 40, 95]. These data confirm that PCR methods could be implemented more systematically to detect other serogroups and *Legionella* species when LD is suspected and when the UAT is negative. To improve the diagnosis rate, more than one method should be implemented if the first test is negative.

Culture of lower respiratory tract specimens is considered the gold standard for LD diagnosis. Even though it is a very demanding test, since it takes several days and requires a complex medium for the pathogen growth, it enables the diagnosis of all *Legionella spp* strains. Patients with more severe infections have much higher bacterial concentrations in their respiratory secretions [1, 2, 23]. The availability of strains allows Whole Genome Sequencing (WGS) of *Legionella*, which is an important tool for identifying the source of infection and for understanding the transmission pathways and mechanisms by which new pathogenic clones emerge [96]. A recent study highlighted the role of WGS in LD surveillance tool as it allows to identify endemic lineages, new clones and to perform phylogenetic analyses for epidemiological purposes [97]. Systematic WGS of Lp is also a powerful tool for first-line high-throughput screening of antibiotic resistance prior to phenotypic validation [98].

## Antimicrobial therapy and patient management

There is a consensus that early clinical diagnosis (< 24 h) and prompt initiation of antibiotic therapy are crucial for the management of the disease and are associated with better outcomes (Table 1). Effective antibiotic therapy against *Legionella spp* depends on the ability of the drug to concentrate in alveolar macrophages, since the alveoli are the primary site of infection in LD [48, 99–102]. Systemic *Legionella*-targeted antibiotic therapy should be included in the empiric regimen for any patients with severe pneumonia. Indeed, Falcone et al. [72] reported that macrolide or levofloxacin within 24 h of hospital admission was protective against clinical deterioration and ICU admission. Dosage are summarized in Table 2.

**Table 1:** Summary of 7 key management recommendations for severe Legionnaire’s disease (LD)

1	Early appropriate antibiotic therapy (within 24 h of hospital admission and 8 h of ICU admission) is associated with better outcomes
2	Use a bactericidal agent with good lung penetration, active against all species causing human infection achieving high intracellular concentrations. Either intravenous levofloxacin or azithromycin are the preferred agents for severe LD. Newer macrolides (clarithromycin, spiramycin) are alternative options
3	Consider combining levofloxacin and macrolides if vasoactive agents are required, for immunocompromised patients or in case of monotherapy treatment failure. Adding rifampin does not appear to improve outcomes but increase adverse events. CS therapy should not be recommended
4	Severe pneumonia often requires above 10 days of therapy and immunocompromised hosts require longer duration than 14 days
5	Utility of procalcitonin in patients with LD is not well-established
6	Consider superinfection, empyema (lung ultrasound assessment) and causes of non-resolving pneumonia if delayed resolution
7	Alert public health authorities to find the source of contamination or in the case of nosocomial cases

**Table 2:** Recommended dosage for severe Legionnaire’s disease

Preferred options	Azithromycin 500 mg IV daily
	Levofloxacin 750 mg IV once daily or 500 mg bid (max)
Alternative options	Clarithromycin 500 mg IV bid
	Spiramycin 3 M UI IV × 3/day

Regarding antibiotic stewardship, the IDSA recommends either a fluoroquinolone or a macrolide as first-line treatment, since they have similar effectiveness for reducing mortality [100]. However, a distinction should be made between severe and non-severe LD. Short courses of therapy are only recommended for non-severe episodes when oral azithromycin is used. For severe patients, intravenous monotherapy with fluoroquinolones or new macrolides (azithromycin or clarithromycin) or a combination therapy of both is recommended. Macrolides other than azithromycin are bacteriostatic against Legionella and appear to be less effective than either azithromycin or levofloxacin. Spiramycin has been used as an alternative in countries, where the intravenous form of azithromycin is not available (for further details we refer the reader to the LD treatment guidelines [1, 100]) or in case of interactions with cyclosporine. With an overall mortality rate of 25–30% among patients requiring mechanical ventilation, it remains controversial whether combination or adjuvant therapy would benefit patients with acute respiratory failure.

In general, infections secondary to intracellular pathogens are slower to respond to antibiotics. Despite effective early therapy, clinical improvement does not appear before 5–7 days. Moreover, LD requires longer courses of treatment so as to ensure cure and to prevent relapse [93]. For immunocompromised hosts, a 21-day course is usually recommended [23, 48, 99, 103]. The usefulness of procalcitonin in LD is not well-established, because its levels do not rise to the same degree as in the case of other pneumonia pathogens. In severe LD we recommend systemic antibiotics, and a switch to oral therapy should be considered when the patient is not receiving vasoactive drugs, respiratory failure is improving, and is able to tolerate the oral route. In non-resolving episodes, lung ultrasound should exclude empyema, in which source control is required. Lung abscess or extra pulmonary infection is rare, but bacterial superinfection should be considered.

Kato et al.’s systematic review and meta-analysis revealed superior effects in terms of mortality and length of hospital stay in patients treated with fluoroquinolones when compared to macrolides [101]. However, a further meta-analysis by Jasper et al. did not find any differences in mortality between the two treatment groups [100]. A recently published systematic review comparing quinolone vs macrolide administration in adult patients with *Legionella* pneumonia found that clinical response and mortality were similar when the two treatments were compared in the global cohort, with a mortality rate of 7.4% [104]. When data from the studies with severe pneumonia were pooled together [68, 102, 105], mortality with quinolones alone was statistically superior to macrolides alone (72.8% vs 30.8%, *p* value 0.02). Other complications and hospital stay were comparable. However, these findings should be interpreted with caution due to the small sample sizes and to the fact that they are based exclusively on heterogeneous observational studies. Further research, in the form of randomised clinical trials, in patients with acute respiratory failure is required to address this gap in our knowledge.

The potential role of combination therapy also remains controversial. On one hand, Cunha et al. suggest that a well-selected monotherapy with azithromycin or a quinolone remains the standard treatment of LD regardless of disease severity [99]. On the other, Chahin et al. recommend administration of a combination therapy of a quinolone plus azithromycin in critically ill patients with severe pneumonia, especially in those with significant comorbidities and in immunocompromised hosts who are refractory to conventional monotherapy regimens [48]. In reference to combined treatments, 25 years ago the association of erythromycin plus rifampicin was recommended for severe *Legionella* pneumonia, in particular in patients with shock, in whom it achieved a significant reduction in mortality, lowered costs, and shortened hospital stays among survivors. The use of rifampicin (or doxycycline) for severe LD is not currently recommended, given the superiority of newer macrolides (e.g., azithromycin, clarithromycin, roxithromycin, and spiramycin) and fluoroquinolones, with more favourable pharmacokinetics and fewer adverse events. On the other hand, in non-severe pneumonia, combined therapy has not proven its efficacy when compared to monotherapy [102]. Among ICU patients, macrolide plus fluoroquinolone combination therapy may be considered in patients with shock, as lower mortality rates were reported in a small cohort of ICU patients with vasoactive drugs than in patients treated with monotherapy [102]. However, due to the current lack of results from clinical trials, further research is required.

As mentioned above, LD has a higher mortality rate in immunocompromised individuals and in those requiring intensive care admission. Because *Legionella* infections induce an intense inflammatory response, steroid therapy or other adjuvant therapies may be appealing alternatives. The role of corticosteroids as an adjuvant therapy in CAP is a hot topic nowadays. A recent meta-analysis reported that hospitalised patients with CAP treated with corticosteroids were less likely to require IMV, but no association was found between corticosteroid therapy and mortality or treatment failure [106]. The ESCAPe randomised clinical trial assessing severe CAP showed that a prolonged administration of a low dose of methylprednisolone did not significantly reduce 60-day mortality [107] but the result may have failed to reach statistical significance merely because of the early termination of recruitment [108]. Evidence is lacking regarding the potential influence of corticosteroid use as an adjuvant steroid therapy for severe LD. In a large multicentre, double-blind, randomised control trial, the administration of hydrocortisone to patients with all-cause severe CAP was shown to reduce the risk of death by day 28 [109]. Although there was a similar distribution of severe LD patients between the two groups (*n* = 22, 5.5% for hydrocortisone therapy vs *n* = 27, 7.3% for placebo), the number of LD was low; no specific conclusion has been proposed for LD. Since prior corticosteroid therapy is a major risk factor for severe LD, and the target should be the macrophages (rather than lymphocytes), its use in patients with LD may lead to either progression or relapse, and so its administration requires great caution.

ICU patients frequently require invasive mechanical ventilation (IMV) and vasopressor use for acute respiratory distress syndrome (ARDS) and acute kidney injury (AKI), which clearly worsen outcome and prognosis [1, 102, 110]. The prevalence of severe ARDS requiring intubation is high, ranging from 50% to 80% in the ICU setting. Most patients develop ARDS and multifocal pneumonia. A high failure rate of non-invasive support (bilevel positive airway pressure ventilation or high-flow nasal cannula) has been reported. In one study, two-thirds of patients receiving a trial of non-invasive ventilation finally required IMV [67]. Some patients with refractory ARDS should be considered for venous–venous extracorporeal membrane oxygenation (ECMO) [67]. A review of survival experience with ECMO for severe LD in a relatively large cohort [52] yielded a survival rate above 70%, a proportion almost identical to the figure (71%) reported by the Extracorporeal Life Support Organization (ELSO). A discussion on management of infections and ECMO is beyond the scope of this manuscript, but updated details have been published elsewhere [111, 112].

Nosocomial infections can be prevented by an adequate environmental management in the hospital setting, since common sources of contaminated water supplies may generate outbreaks [48]. The need to perform a microbiological evaluation and the initiation of empiric antibiotic therapy for *Legionella* spp in all cases of severe CAP are well-established in the current guidelines. Nonetheless, empiric treatment of *Legionella* is not recommended in the initial therapy of nosocomial-acquired pneumonia, except in institutions with persistent endemic episodes [21]. However, it has been demonstrated that in patients with *Legionella*, a nosocomial source of infection is independently associated with increased 30-day mortality [103].

## Antibiotic resistance and antimicrobial susceptibility testing

Fortunately, antibiotic resistance is not yet a problem for *Legionella* infections; *Legionella* are susceptible to the antibiotics commonly used in therapy (macrolides, fluoroquinolones and rifampicin). Very occasionally, however, relapses or recurrences of LD in correctly treated patients have been reported [93, 94]. The lack of international guidelines and commercially available tools limit the performance of systematic antimicrobial susceptibility testing in *Legionella* [113]; it may be recommended in specific contexts, such as slowly resolving or non-resolving infections.

Several studies have demonstrated the ease with which antibiotic-resistant mutants can be selected in vitro, and have identified the molecular mechanisms involved [114–116]. In contrast, the absence (or near absence) of human-to-human transmission and of healthy carriers described to date does not favor the emergence of in vivo* Legionella* resistant mutants. To date, only one fluoroquinolone-resistant clinical Lp1 strain has been described in an infection setting, in 2014 [117]. In that report, it was not clear whether the resistant mutant was selected in the patient during antibiotic therapy or whether the patient was infected upstream by a resistant environmental strain. In vivo selection of fluoroquinolone resistance mutations in Lp has been reported in two other infected patients, with an increasing proportion of mutant copies during fluoroquinolone therapy, using targeted next-generation sequencing. The first highly macrolide-resistant Lp1 strain was recently isolated from the water system of a hotel during the investigation of a case of LD [98]. As the patient was treated with a fluoroquinolone, the macrolide resistance did not affect the clinical course. More than the concern about therapeutic failures caused by this still exceptional resistance, this detection raises the question of the environmental factors inducing the acquisition and the spread of these resistant strains. Nevertheless, this description shows the value of systematic screening of antimicrobial resistance in both environmental and clinical settings using WGS, as the sequencing of bacterial strains has increased in recent years.

## Areas for future research

The incidence of LD has increased in many countries in recent years. This is due probably to the constant development of water systems linked to human activities, which are important factors in the multiplication of this bacterium and are powerful vectors; moreover, certain meteorological conditions such as heat and rainfall are associated with an increase in the incidence of LD. Consequently, studies investigating new sources of infection are now needed [118]. Finally, smoking, age and immunosuppression are major risk factors for LD. The ageing and the development of immunosuppressive treatments must also be taken into account. Despite its rising incidence, LD is probably still under-diagnosed, especially in cases of non-serogroup 1 Lp and *L.* non-*pneumophila* infections. The use of PCR targeting Lp and *L.* non-*pneumophila* in ICU patients with pneumonia needs to be increased. Despite a well-administered antibiotic therapy and the absence of antibiotic resistance, treatment failure and mortality in the ICU remain high. There is a clear need for randomised clinical trials in this setting comparing different regimens and combination therapy [104]. Moreover, a dysregulated immune response with an intense pro-inflammatory phase and immunoparalysis appears to lead to poor clinical outcomes. Further studies evaluating the immune response in clinical trials identifying different immune-phenotypes [45, 108, 119] are needed to assess the value of personalised treatment as an adjunct to antibiotic therapy. It would also be important to examine the effects of hydrocortisone on outcomes in patients with severe LD.

## Conclusion

In conclusion, as previously discussed, numerous factors influence the mortality rate of LD, including ICU admission, the underlying immune status, and the nosocomial source of the infection. The host immune response (hyperinflammation and/or immunoparalysis) may also be associated with increased severity. As the incidence of LD is rising, studies of specific biomarkers of severity may be of great interest. Diagnostic methods for LD have improved with the widespread use of UAT and the development of PCR methods that can detect all *Legionella* species. Therapy is based on macrolides, quinolones or a combination of the two, with a prolonged duration in severe cases. Further studies comparing different regimens and/or evaluating host-directed therapies are now needed. In severe cases we prefer to use prolonged systemic therapy with newer macrolides, levofloxacin, or a combination of both. Randomised clinical trials comparing safety and efficacy of different regimens and host-directed therapies in severe LD are also warranted.

## Data Availability

Not applicable.

## Abbreviations

APACHE II: Acute Physiology And Chronic Health Evaluation
AST: Antibiotic susceptibility testing
ATS: American Thoracic Society
CAP: Community-acquired pneumonia
DCs: Dendritic cells
DNA: Deoxyribonucleic Acid
ECMO: Extracorporeal membrane oxygenation
FlaA: Flagellin
ICU: Intensive care unit
IDSA: Infectious Diseases Society of America
IL: Interleukin
IMV: Invasive mechanical ventilation
LD: Legionnaires’ disease
Lp: *Legionella pneumophila*
LPS: Lipopolysaccharide
Mip: Macrophage infectivity potentiator
MV: Mechanical ventilation
NAIP5: NLR family, apoptosis inhibitory protein 5
NF-κB: Nuclear Factor kappa-B
NK: Natural killer
NOD: Nucleotide Oligomerization Domain
PCR: Polymerase Chain Reaction
PNNs: Polynuclear neutrophils
ProA: Zinc metalloprotease
RIG-I: Retinoic Acid Inducible Gene I
SAPS II: Simplified Acute Physiology Score
T4SS: Type IV secretion system
TLRs: Toll-like receptors
TNF-α: Tumor necrosis Factor-alpha
UAT: Urinary antigen tests
US: United States
WGS: Whole Genome Sequencing
